# Assessment of the Educational Needs of Spanish Nurses in the Care and Management of Patients with Diabetes

**DOI:** 10.3390/healthcare13050526

**Published:** 2025-02-28

**Authors:** Guadalupe Fontán-Vinagre, Carlos Ruíz-Núñez, Silvia Domínguez-Fernández, Diego Ayuso-Murillo, Ivan Herrera-Peco

**Affiliations:** 1 Spanish Institute for Nursing Research, C. de la Sierra de Pajarejo, 13, Moncloa-Aravaca, 28023 Madrid, Spain; g.fontan@ieinstituto.es (G.F.-V.); s.dominguez@ieinstituto.es (S.D.-F.); 2 Unidad de Innovación, Centro de Emergencias Sanitarias 061, 29590 Málaga, Spain; carlos.ruiz.nunez.sspa@juntadeandalucia.es; 3 Consejería de Familia, Juventud y Asuntos Sociales de la Comunidad de Madrid, C. de Espartinas, 10, 28001 Madrid, Spain; 4 Consejo General de Enfermería, Calle Fuente del Rey 2, 28023 Madrid, Spain; 5 Socialhealthcare—UAX Research Group, Faculty of Health Sciences, Universidad Alfonso X el Sabio, Villanueva de la Cañada, 28691 Madrid, Spain; 6 Faculty of Health Sciences—HM Hospitals, University Camilo José Cela, Urb. Villafranca del Castillo, 49, Villanueva de la Cañada, 28692 Madrid, Spain; 7 Instituto de Investigación Sanitaria HM Hospitales, 28015 Madrid, Spain

**Keywords:** diabetes, nursing education, continuing professional development, hybrid learning, training barriers, clinical competencies

## Abstract

**Introduction:** The rising prevalence of chronic diseases such as diabetes poses significant challenges to healthcare systems globally, requiring specialized care and management. Nurses play an essential role in educating and caring for patients, but current continuing education programs often fail to meet the practical needs of clinical settings. **Methods:** This study used a qualitative descriptive–interpretative approach, conducting semi-structured interviews with 24 nurses working in primary care and hospital settings across Spain. The interviews focused on their experiences, perceptions, and educational needs in diabetes care. A thematic analysis was performed to identify key trends and insights. **Results:** Nurses emphasized the importance of ongoing education in building professional confidence and improving patient care outcomes. However, they reported significant barriers, including limited time, high costs, and a lack of practical focus in existing training programs. Participants favored hybrid learning models, which combine the flexibility of online training with the hands-on experience of in-person sessions. Nurses in rural areas highlighted the value of virtual training to overcome geographic constraints, while those in urban environments preferred hybrid approaches. Additionally, nurses called for accessible and concise resources, such as digital libraries and clinical simulations, to support decision making in real time. **Conclusions:** To be effective, diabetes- and ostomy-focused continuing education must align with nurses’ clinical realities and individual needs. Combining digital tools with practical in-person learning can enhance accessibility and foster the practical application of skills. These findings provide actionable insights for designing education programs that advance both patient care and nurses’ professional development.

## 1. Introduction

Nowadays, non-communicable chronic diseases pose a significant challenge to global health systems due to their high prevalence and the demands of ongoing patient management [1,2]. Among these, diabetes is particularly demanding in terms of specialized healthcare [3,4].

Diabetes is recognized as a chronic disease with a considerable impact on global public health. It is projected that, by 2030, it will be classified as the seventh leading cause of death worldwide [5]. Currently, in Spain, there is a notable increase in the prevalence of this disease, resulting in a growing demand for specialized health services and an increase in hospitalization rates [6]. Internationally, the burden that diabetes places on health systems is substantial, particularly in middle- and high-income countries, where associated complications such as retinopathy, neuropathy, and diabetic nephropathy are common and require specialized care [7].

The complexity of diabetes, as a chronic disease, necessitates proper management of the care provided by healthcare professionals, making it crucial to have healthcare professionals who can serve as both educators [6,8,9,10,11,12] and supporters for patients managing the disease [13,14,15]. Nurses, as essential members of multidisciplinary healthcare teams, play a critical role in managing this chronic condition. Their ability to provide individualized patient-centered care leads to increased patient knowledge, early detection of behavioral changes, and improved clinical outcomes [1,2]. Furthermore, they are crucial in encouraging patients to adopt healthy lifestyle habits [2,3], essential steps in managing chronic conditions like diabetes [3,7]. However, it is important to recognize that, to serve as reliable educators for patients with diabetes, nurses must receive training that is tailored to the specific demands of their professional practice [4]. Continuing education, often facilitated by healthcare institutions, is not merely a supplement to routine care but a critical element in improving patient outcomes and minimizing long-term complications associated with diabetes [5,7,8]. Such focused educational programs empower nurses to conduct thorough assessments, develop effective care plans, implement appropriate nursing interventions, and critically evaluate patient outcomes [5,9,12,15], thereby directly contributing to a reduction in the broader socioeconomic challenges posed by these chronic diseases [7]. By improving nursing care through education, healthcare systems can alleviate some of the economic pressures of diabetes management, which include not only direct medical costs but also indirect costs from lost productivity and disability [8].

It is essential to view continuing education as a key element of the nursing profession, as it ensures the ongoing development of knowledge and skills. This type of training has been shown to improve patient care outcomes [9], as nurses who engage in regular education are better equipped to implement evidence-based practices and adapt to technological advancements [10,11]. The direct correlation between continuing education and enhanced patient outcomes emphasizes the importance of such programs in professional practice. In-depth studies focusing on this correlation can provide further insights into how structured educational interventions lead to better healthcare delivery [16]. Although in some countries, such as Canada and Australia, continuing education is mandatory for maintaining nursing licensure, emphasizing its importance in professional practice [6,12], this regulatory approach highlights the recognized value of ongoing professional development in maintaining high standards of nursing care. Conversely, in Spain, while there are guidelines highlighting the need for ongoing education, it is not a formal requirement for maintaining the ability to practice as a nurse [13]. This difference in regulatory frameworks can serve as a basis for comparative studies that evaluate the impact of mandatory versus voluntary continuing education on nursing practice outcomes [17].

The value of continuing education lies in its design being focused on ensuring that the knowledge provided can be effectively integrated into clinical practice. However, the lack of practical application is often identified as a limitation of such training programs [14]. Studies that explore the design and implementation of continuing education programs, and their effectiveness in integrating theoretical knowledge with practical skills, could provide valuable insights into improving these programs [18,19] Enhancing the practical component of continuing education may involve the use of simulations, case studies, and real-life scenario analyses, which have been shown to significantly improve clinical competencies [15,17,20]. To address this issue, various methods focused on in-person learning have been proposed. For instance, informal workplace-based learning approaches that involve interaction with colleagues are highly valued by nurses [15]. Additionally, studies highlight blended learning as an effective option for knowledge retention, while also fostering the development of skills and competencies among nurses [18,19]. These approaches have proven particularly applicable in the care of chronic illnesses, such as diabetes [21].

Within the field of continuing education, virtual or online training has seen significant growth, particularly since the COVID-19 pandemic, and continues to be widely utilized today [12,22]. This format offers numerous advantages, such as flexibility in accessing and consuming content [23], better work–life balance, and the ability to learn independently of material resources. It also allows for the standardization of content and reaches broader, even global, audiences [24]. These benefits align well with the realities of clinical environments, where time is often limited. However, online training also presents challenges, such as limited face-to-face interaction, varying levels of digital literacy among nurses [22], and the complexity of some learning platforms [25]. In this context, training specifically focused on diabetes management should be a priority in continuing education programs, as this disease remains one of the greatest challenges for healthcare systems worldwide [24,25].

This study aims to identify and understand the training needs of nurses who care for patients with diabetes, exploring both the topics they find relevant and the competencies they need to develop or update for comprehensive diabetes care. Additionally, it seeks to assess nurses’ preferences regarding the delivery methods of this training, examining their inclinations toward in-person, remote, or hybrid formats. Using a qualitative approach, this study aims to provide a detailed understanding of nurses’ training expectations and demands to design accessible and effective continuing education programs tailored to clinical environments and technological advancements in diabetes care.

## 2. Materials and Methods

### 2.1. Study Design

This study employed a qualitative descriptive–interpretative approach to explore the perceptions and experiences of practicing nurses regarding training and related resources for managing and treating patients with diabetes in both hospital and primary care settings. Semi-structured interviews were used to gain a detailed understanding of nurses’ perspectives and lived experiences. These interviews provided insights into their approaches to diabetic patient care, the training they had received, and areas for improvement in continuing education.

### 2.2. Participants

The sample consisted of 24 nurses recruited nationally by the Nursing Research Institute. Participants were selected through purposive sampling to ensure the inclusion of nurses from various care settings (hospital and primary care). Inclusion criteria were as follows: (i) being a licensed nurse actively practicing in hospital or primary care settings and (ii) having experience managing patients with diabetes or an ostomy. Nurses without direct contact with these patients or those not actively practicing during the study period were excluded.

Participant recruitment continued until data saturation, in qualitative research, was achieved, defined as the point at which additional interviews no longer provided new analytical insights [26]. This size was sufficient to ensure a comprehensive exploration of diverse experiences and thematic analysis.

### 2.3. Data Collection Procedure

Data was collected through semi-structured interviews conducted between January and June 2024. The interviews were held via videoconference, depending on participants’ availability and preferences. A previously validated interview guide was used to explore the following topics: (i) participants’ perceptions of caring for patients with diabetes and the training they had received; (ii) how they addressed uncertainties and whom they consulted when needed; (iii) the type of continuing education they had received and wished to receive; and (iv) their views on which type of training would be most effective in their specific context.

Data collection continued until saturation was reached, defined as the point at which participants no longer provided new information. The interviews lasted an average of 58 min, ranging from 40 to 118 min. Audio recordings were made of all interviews, which were then fully transcribed for subsequent analysis.

### 2.4. Data Analysis

The transcribed interviews were analyzed using the thematic analysis method outlined by Braun and Clarke [27]. The process included the following steps: (i) familiarization with the data, (ii) generation of initial codes, (iii) identification of themes, (iv) review of themes, (v) definition and naming of themes, and (vi) preparation of the final report. The transcripts were not shared with participants for verification, as this study aimed to focus on their initial perceptions without subsequent influence.

### 2.5. Ethical Considerations

This study received approval from the Research Ethics Committee of Universidad Alfonso X el Sabio (approval code: 2023_04/202 date: April 2023). Written informed consent was obtained from all participants prior to the interviews, ensuring data confidentiality and anonymity. The interviews were recorded and anonymized before transcription.

## 3. Results

### 3.1. Sample Description

The sample in this study consisted of nursing professionals specializing in the care and management of patients with diabetes. Participants were selected from various regions across Spain, including Andalusia, Aragon, the Canary Islands, Castile and León, Catalonia, Extremadura, Madrid, Murcia, and the Basque Country. The selection process ensured a diverse representation of professional profiles and care settings related to the treatment of individuals with this chronic condition.

In terms of age, the sample was divided into two distinct groups: nurses under 40 years old (*n* = 12) and those over 40 (*n* = 12). Additionally, the participants were grouped based on their care settings. In primary care, 6 nurses were over 40 years old and 5 were under 40 years old. In specialized care, 6 nurses were under 40 years old, and 7 nurses were under 7 years old. This distribution allowed this study to capture a wide range of experiences, spanning both outpatient and hospital-based care.

### 3.2. Main Topics of This Study

The analysis of the topics covered in this study identified the following key areas: (i) perceptions of training needs, (ii) motivations for pursuing continuing education, and (iii) barriers and facilitators to accessing specialized training. Additionally, previous training experiences were explored, focusing on their impact on clinical practice and the evaluation of available training resources. Lastly, proposals were discussed for designing training tools tailored to the specific needs of the nursing workforce, with particular emphasis on format preferences (in-person, online, hybrid) and essential features for digital training support tools.

Three main themes emerged from this study. The first theme addressed training needs and motivations for continuing to develop competencies in diabetes management. The second focused on barriers to accessing training, including external factors and attitudinal challenges. The third theme examined preferences for training modalities, highlighting the value of hybrid approaches that combine theoretical and practical components.

A fourth theme also emerged, centered on the characteristics of the training demanded, emphasizing the importance of access to concise specific information. In total, seven subcategories were generated from the participants’ shared experiences, which were organized under the main themes identified (Table 1).

#### 3.2.1. Needs and Motivations About Continuing Training

Continuing formation was perceived as a fundamental necessity to ensure the ongoing update of both practical and theoretical knowledge while strengthening professional autonomy. Participants emphasized the importance of training in improving the quality of care and enhancing professional empowerment.

They highlighted the need to refresh knowledge as an essential requirement for ensuring patient safety and maintaining high standards of care. This perspective aligns with the category of continuous updating, where training is seen as an indispensable tool for addressing the challenges of clinical practice. Staying current with scientific and technological advancements was identified as a key strategy to avoid professional obsolescence and effectively respond to modern healthcare demands (Table 2).

Professional autonomy and quality of care emerged as closely connected aspects of professional development. Participants noted that increased knowledge provides them with confidence and security, translating into stronger decision-making abilities, as could be seen in this verbatim: “Solid training allows you to act with confidence in critical situations” (Table 2). This perspective falls under the category of safety and quality, emphasizing that training not only enhances technical competence but also supports more autonomous and decisive practice (Table 2). Participants pointed out that improving their skills enables them to deliver more effective patient-centered care, thereby enhancing their sense of control over their professional practice.

Lastly, a widespread concern was noted regarding the lack of a practical focus in the training they had received, which hindered the immediate application of knowledge in clinical settings. This perspective was categorized under limited applicability, as participants expressed that many training programs prioritize theoretical content over practical application. Applied training was considered essential to improve technical skills, such as managing medical devices and specific procedures in diabetes care. Additionally, the inclusion of workshops and simulations in training programs was highlighted as crucial to fostering a more dynamic and useful learning experience for daily practice (Table 2).

#### 3.2.2. Barriers

When evaluating potential barriers to training, participants identified two main types: external and internal barriers.

As an external barrier, insufficient time was highlighted as the primary obstacle to accessing training, represented by verbatims like “Time is the biggest issue. You can’t always attend courses outside work hours”. High workloads, rotating shifts, and the need to balance work and personal life were repeatedly mentioned, with many participants noting that training often must be completed outside of working hours, posing an additional challenge. This issue falls under the category of limited availability, reflecting the difficulty of finding the appropriate time for learning. Participants also emphasized the feeling of being overworked, which discourages the extra effort required to attend training sessions, particularly those requiring physical attendance (Table 3).

Economic cost was mentioned as another external barrier. Participants reported comments like “If you want good training, you have to pay for it, and it’s not always affordable”, related to specialized courses and programs being self-funded, adding financial strain, especially for early career professionals. This issue was categorized as personal investment, highlighting that high-quality training, whether in-person or online, often involves substantial costs. While some participants viewed this expense as necessary for professional growth, others found the cost to be a deterrent, limiting access to more advanced or specialized programs.

At an internal level, resistance to change emerged as an attitudinal barrier, hindering professional development and the adoption of new practices. Some participants noted a culture of complacency in certain workplaces, where traditional methods are favored over continuous learning. This issue falls under the category of complacency, characterized by a lack of intrinsic motivation to seek new learning opportunities, as could be seen in comments like “Sometimes it’s easier to stick with what we already know instead of learning something new”. Additionally, this phenomenon was more common among older and more experienced professionals, who, in some cases, expressed skepticism toward new technologies and training methods, such as online or hybrid formats. This attitude perpetuates a static approach to improving skills to clinical practice (Table 3).

#### 3.2.3. Training Modalities and Preferences

Participants highlighted that the choice of training modality was a key factor in improving both access to education and adherence to continuing education programs focused on diabetic patient care. While discussing the advantages of in-person training, it is essential to consider the perspectives of frontline professionals who utilize these sessions to enhance their skills, for example, with comments like “Nothing beats the immediate feedback and hands-on experience of in-person training sessions. When I learned how to use a new insulin pump, being able to practice on a simulator with an instructor right there made all the difference”. Within the category of superior quality, participants emphasized that in-person sessions facilitate experiential learning by allowing direct interaction with instructors and training materials. Additionally, this format fosters an environment where questions can be addressed, and hands-on exercises can be performed by factors considered essential for mastering specific skills, such as operating medical devices and advanced techniques.

Online training, on the other hand, was positively rated for its flexibility and accessibility. This training modality offers unmatched flexibility, which is crucial for nurses operating in less accessible regions, comments such as “I prefer online courses because I can access them whenever I have some free time, which is often late at night or early in the morning. This flexibility is crucial for those of us in rural areas where travel to training centers is not feasible” explain why this modality suits the needs of some nurses. Under the category of flexibility and reach, participants acknowledged that this modality enables learning to be adapted to individual schedules, making it easier to balance work and family responsibilities. Furthermore, remote access eliminates geographical barriers, allowing participation in programs that would otherwise be inaccessible due to location. However, some participants noted that online training is less effective for developing practical skills, emphasizing the need to complement it with in-person sessions.

Finally, hybrid training was identified as the most suitable option to meet current educational needs. Within the category of best combination, participants highlighted that this format combines the flexibility of online training with the practical depth of in-person sessions. They particularly valued its ability to deliver theoretical content virtually while reserving in-person sessions for practical activities and simulations. The hybrid model, combining online and in-person elements, is increasingly favored among nursing professionals, which could be observed in verbatims like “Hybrid models are the future of nursing education. They combine the convenience of online learning with crucial hands-on sessions where you can apply what you’ve learned in a practical setting”.

The analysis revealed differences among participants regarding online training, depending on whether they worked in rural or urban areas. Nurses in rural settings emphasized the need to prioritize online training as a key strategy to overcome geographical barriers and improve access to educational programs. This preference was attributed to travel limitations and the reduced availability of in-person training in these areas, making digital resources essential for their continuous professional development (Table 4).

In contrast, nurses working in urban settings showed a stronger preference for hybrid training, appreciating the combination of virtual sessions with in-person practice. This group acknowledged the flexibility offered by online training but also emphasized the importance of in-person sessions for developing practical skills and reinforcing applied learning.

#### 3.2.4. Characteristics of Desired Training

The findings revealed that nurses participating in this study identified key features that continuing education programs should incorporate to better align with their professional needs and care settings. One of the suggestions included creating digital repositories accessible via mobile devices, something observed in the following verbatim: “We need quick access to updated protocols and procedures, especially in emergency situations. A digital repository accessible from any device would be a game-changer for us”. These resources would allow quick access to procedures, updated protocols, or practical recommendations whenever questions arise during clinical practice. Participants noted that such tools would facilitate real-time decision making, particularly in critical situations. They also suggested that this type of training should ideally be offered by professional nursing organizations (Table 5).

Additionally, the demand for concise and relevant training content was high among nurses who juggle multiple responsibilities, observed in comments like “Short, focused training modules, like video tutorials on patient care techniques, help me stay updated without overwhelming my schedule. These should be clear and to the point”. The development of concise and focused content, such as short educational videos (1–3 min) and podcasts, was recommended (Table 5). These formats were appreciated for their ability to provide precise applicable information within a limited timeframe, making them suitable for irregular schedules and the demands of nursing work. Participants highlighted the value of videos featuring practical demonstrations of specific techniques, such as insulin administration, insulin pump management, or continuous glucose monitoring device handling.

Finally, participants emphasized the importance of including downloadable materials and quick reference guides in training programs, designed for immediate consultation in clinical settings (Table 5). These resources, along with virtual simulations and interactive case studies, were perceived as essential tools to strengthen the acquisition of practical skills and boost confidence in clinical decision making, something highlighted in verbatims like, Downloadable quick-reference guides and virtual simulations would help us apply new knowledge immediately, which is often when it’s most needed”.

## 4. Discussion

The present study focuses on exploring the training needs of Spanish nurses tasked with providing care and clinical management for patients with chronic diseases diagnosed with diabetes and patients with ostomies. To the best of the authors’ knowledge, this is the first study to deeply examine both the specific training demands and the barriers to accessing such education, aimed at improving clinical practice in caring for these kinds of patients.

A crucial aspect that warrants further exploration is how demographic and professional variables such as age, experience, and the work setting of participants influence their perceptions and experiences of continuing education. It is well recognized that younger generations of nurses may be more familiar with and receptive to digital learning formats, given their upbringing with technology [10,11]. Conversely, more experienced nurses, who may have established learning practices throughout their careers, might prefer more traditional methods of continuing education [12]. These generational differences could have significant implications for designing educational programs that are effective and appealing to all nursing staff.

First, it is essential to highlight that continuing education is a key element of professional development for nurses [9] and plays a critical role in effective diabetes management [9,10]. This aligns with prior research emphasizing the key role of specialized training in managing chronic conditions [4]. Participants in this study underscored the importance of continuously updating their knowledge, noting its positive impact on both professional autonomy and patient safety. These findings are consistent with the existing literature that identifies continuing education as a pivotal factor in the adoption of evidence-based practices [9].

However, the findings also revealed significant barriers to accessing this training, stemming not only from external factors, such as time management and financial costs, but also from attitudinal aspects that limit interest in continuous learning. Regarding external factors, the results of this study align with the prior research that identifies workload and work–life balance challenges as recurring obstacles to continuing education for healthcare professionals [12,14]. This study reinforces these observations by demonstrating that nurses working in specialized care settings face greater constraints due to the need to develop advanced practical skills in diverse areas such as wound care, ostomies [28], surgical care [29], and emergency care [30], along with diabetes management [31]. These demands often require additional time and in-person resources, making access to training even more challenging for these professionals.

Regarding training modalities, the analysis revealed significant differences based on the care context. Primary care nurses in rural settings expressed a strong preference for online training due to geographical and travel challenges. This finding aligns with previous studies highlighting the benefits of virtual education in overcoming logistical barriers and expanding access to educational programs [12,14]. Conversely, nurses in urban and specialized care settings showed a greater inclination toward hybrid models, combining online training for theoretical content with in-person sessions for developing practical skills. This supports research suggesting that blended learning enhances knowledge retention and facilitates practical application [17,18]. Additionally, it could be observed that attitudes toward continuing education may vary depending on the care context. Nurses in rural areas emphasized the need for flexible training formats to accommodate time and travel constraints, consistent with the research showing the positive impact of online education on accessibility and work–life balance [19,20]. However, concerns about the quality of virtual training were also identified, particularly regarding the development of practical skills. This underscores the importance of designing hybrid programs that effectively integrate theoretical knowledge with hands-on practice [16,32]. Resistance to change and complacency among some professionals were identified as attitudinal barriers that hinder the adoption of new training modalities. This finding aligns with studies highlighting professional inertia as a key factor that obstructs knowledge updates [22]. Overcoming these barriers requires targeted strategies, such as integrating collaborative learning programs into the workplace, which have proven effective in promoting continuous professional development [15,33].

Finally, there is a clear need to tailor continuing education programs to the specific demands of nurses, emphasizing the value of accessible and dynamic tools to enhance clinical practice. Previous studies have noted that digital technologies, such as information repositories and mobile resources, facilitate constant knowledge updates and quick problem solving in clinical settings [20,21]. Additionally, brief formats like videos and podcasts have been shown to optimize autonomous learning, particularly in professions with high workloads [17,18]. The growing preference for hybrid models illustrates a significant trend towards blending virtual tools with in-person training, combining flexibility with hands-on practice [16].

To further address the challenges highlighted by this study and enhance the practical applicability of our findings, innovative solutions such as gamification and virtual mentoring should be integrated into training programs. Gamification can transform learning into a more engaging and interactive experience [34], which is particularly effective in sustaining the interest and participation of nurses. Implementing game-based learning strategies could involve the use of virtual simulations that mimic real-life scenarios, allowing nurses to gain practical skills in a controlled, competitive, and fun environment [35]. This approach not only enhances learning outcomes but also increases motivation and engagement among learners.

Similarly, virtual mentoring can provide personalized support and guidance, leveraging the expertise of seasoned professionals who can share insights and feedback through digital platforms. This method allows for real-time interaction educational experiences that can adapt to the individual learning pace and style of each nurse [35,36]. Virtual mentoring programs can be particularly advantageous in rural settings [37], where physical access to seasoned mentors may be limited [38].

This study presents certain limitations that should be considered when interpreting the results. While the qualitative design allowed for an in-depth exploration of nurses’ perceptions and experiences, the findings cannot be generalized to other care contexts or regions. Additionally, the use of semi-structured interviews may have been influenced by social desirability bias, potentially affecting the spontaneity of responses. Despite these limitations, this study offers significant strengths. One of its primary contributions is identifying differences in training needs based on the type of care and the healthcare setting, providing valuable insights for designing programs tailored to specific contexts. Furthermore, it offers a detailed analysis of the most effective training modalities, highlighting the utility of online training in rural areas and the suitability of hybrid models in urban and specialized care settings.

## 5. Conclusions

This study highlights how crucial ongoing education is for nurses caring for patients with diabetes. It plays a key role in keeping their knowledge up to date, building practical skills, and improving the quality of care. The findings show that continuing education is essential for patient safety and helps nurses gain more professional independence, especially when managing this chronic condition. However, major barriers like time constraints, financial costs, and resistance to change make it clear that training programs need to be more flexible and accessible, especially in high-workload or low-resource settings.

This study also finds differences in training preferences. For example, primary care nurses in rural areas tend to prefer online education since it helps them overcome geographic challenges. On the other hand, nurses in urban and specialized settings favor hybrid models that mix online theory with in-person practice. This shows why training programs need to be tailored to fit different healthcare environments.

To close the gap between current training and the evolving demands of healthcare, continuing education programs need some of the following key improvements: (i) Gamification, which makes learning more engaging and interactive, boosting participation and knowledge retention. Adding competitive elements and rewards can make training more effective and enjoyable. (ii) Virtual mentoring, which gives nurses real-time support and personalized guidance from experienced professionals. This is especially useful in rural areas, where traditional mentorship opportunities may be limited. (iii) Implementing hybrid training model—combining the flexibility of online learning with the hands-on experience of in-person sessions—can help meet the diverse learning needs of nurses across different settings. Making training modules easily accessible on multiple devices, including smartphones, would also allow nurses to apply what they learn right away in real-world situations. (iv) Lastly, including a continuous feedback loop in training programs can help adjust content to meet the changing needs of nurses. Engaging policymakers to push for mandatory continuing education, as in countries like Canada and Australia, could further reinforce the importance of ongoing professional development.

Together, these strategies aim to improve nurse education and, ultimately, patient care across various healthcare settings. By addressing current gaps with innovative, accessible solutions, continuing education can become more effective, ensuring that nurses are fully prepared to meet the challenges of modern healthcare.

## Figures and Tables

**Table 1:** Main themes and categories identified in this study on specialized nursing training in diabetes.

Main Themes	Categories
Training Needs and Motivations	- Continuous updating.
	- Professional autonomy and quality of care.
	- Lack of a practical focus.
Barriers to Training	- Limited availability.
	- Financial costs.
	- Resistance to change.
Training Modalities and Preferences	- Superior quality (in-person training).
	- Flexibility and accessibility (online training).
	- Best combination (hybrid training).
Characteristics of Desired Training	- Quick access to information (digital repositories).
	- Brief and dynamic content (short videos, podcasts).

**Table 2:** Needs and motivations.

Category	Code	Verbatims
Continuous Updating	“Ongoing education”	“People’s lives depend on you, so you have to keep learning.” “Continuing education should be mandatory to stay up to date.” “With rapid technological changes, it’s easy to fall behind if you don’t keep learning constantly.”
Safety and Quality	“Professional autonomy”	“Knowledge gives you more autonomy and confidence.” “Solid training allows you to act with confidence in critical situations.” “The more you learn, the more confident you feel, and the better you can help patients.”
Limited Applicability	“Lack of practical focus”	“Often, practical skills are learned by handling devices.” “We need practical training that we can apply the next day at work.” “Sometimes courses are too theoretical, making it hard to apply what you’ve learned in a clinical setting.”

**Table 3:** Barriers to training.

Category	Code	Verbatims
Limited Availability	“Lack of time”	“Evenings, weekends… everyone manages as best they can.” “Time is the biggest issue. You can’t always attend courses outside work hours.” “Between work and personal life, there’s barely any time for training.”
Personal Investment	“Economic cost”	“Training costs money, especially quality programs.” “If you want good training, you have to pay for it, and it’s not always affordable.” “Not all affordable training is useful; the best ones tend to be expensive.”
Complacency	“Resistance to change”	“We’re used to working, clocking out, and forgetting about work.” “Some people prefer to keep doing things the same way, even if it’s not the best.” “Sometimes it’s easier to stick with what we already know instead of learning something new.”

**Table 4:** Training preferences based on care type and setting.

Care Setting	Category	Verbatims
Rural	Need to enhance online training	“In rural areas, it’s difficult to travel for in-person courses; we need more online options.” “Online training allows us to access updated content without spending time on long commutes.”
Urban	Preference for hybrid or in-person training	“Online training makes it easier to organize, but I think it should be combined with practical in-person sessions.” “In the city, we have more in-person options, but hybrid formats balance theory and practice.” “In the hospital, we need to learn specific techniques that require hands-on, in-person training.”

**Table 5:** Key features of desired training tools.

Category	Code	Verbatims
Quick Access to Information	Accessible digital repositories	“It would be very helpful to have a digital repository where I can check updated protocols on my phone anytime.”
		“When I have a question during practice, I need quick access to information; a mobile app would solve that.”
		“A centralized digital resource would make it easier to access techniques and procedures in critical situations.”
Brief and Dynamic Content	Short videos and podcasts	“A three-minute video on insulin pump management would be perfect for resolving doubts while I’m working.”
		“Podcasts allow me to learn during my commute or breaks; it’s quick and accessible information.”
		“Practical videos should be short but very visual to quickly learn specific techniques.”
Downloadable Materials and Quick Guides	Resources for immediate consultation	“Having downloadable guides would make my work easier because I could review them even without internet access.”
		“In critical situations, I need to quickly reference a procedure; a downloadable pocket guide would be ideal.”
		“Virtual simulations would help me practice procedures before applying them to real patients.”

## Data Availability

The raw data supporting the conclusions of this article will be made available by the authors on request.
